# PD12 - Living on a farm protects from allergic rhinitis at school age

**DOI:** 10.1186/2045-7022-4-S1-P12

**Published:** 2014-02-28

**Authors:** Bernt Alm, Emma Goksör, Nils Åberg, Per Möllborg, Rolf Pettersson, Laslo Erdes, Göran Wennergren

**Affiliations:** 1 Department of Pediatrics, Institution of Clinical Sciences, University of Gothenburg, Gothenburg, Sweden; 2 Central Infant Welfare Unit, Fyrbodal Health Care Region, Uddevalla, Sweden; 3 Pediatric Outpatient Clinic, Södra Älvsborg Hospital, Skene, Sweden

## Background

Family history plays a major role in the development of allergic rhinitis. External influences, such as a farm childhood and fish introduction have been suggested to play a protective role. The aim was to analyse early risk factors and protective factors for allergic rhinitis at school age.

## Methods

The material is a prospective, longitudinal study of a cohort of children born in the region of western Sweden in 2003 where 8,176 families (50% of the birth cohort) were randomly selected. The parents answered questionnaires at 6 months, 12 months, 4½ years and 8 years of age. The response rate at eight years was 80% (4,051 of 5,044 questionnaires distributed).

## Results

At eight years of age, 441 children (11.3%) had used medicines for allergic rhinitis the past twelve months. The mean onset age was 5.1 year and 61.9% were boys. In a multivariate analysis of factors associated with allergic rhinitis with p<0.1, we found that living on a farm at 4½ years was inversely associated with allergic rhinitis treated with medicines at 8 years (adjusted odds ratio 0.31, 95% confidence interval (0.13, 0.78)). Positive associations were seen with parental allergic rhinitis (2.73 (2.12, 3.52)), food allergy first year (2.45 (1.61, 3.73)), eczema first year (1.97 (1.50, 2.59)), neonatal antibiotics (1.75 (1.03, 2.97)) and male gender (1.35 (1.05, 1.74)).

## Conclusion

In conclusion, we found that a family history of rhinitis, early food allergy, early eczema and male gender increased the risk of rhinitis at school age. Furthermore, we found a protective effect of living on a farm at preschool age, and that antibiotics neonatally increased the risk. Both findings are compatible with the hygiene hypothesis.

